# Effects of prehospital adrenaline administration on out-of-hospital cardiac arrest outcomes: a systematic review and meta-analysis

**DOI:** 10.1186/s13054-014-0463-7

**Published:** 2014-07-31

**Authors:** Pongsakorn Atiksawedparit, Sasivimol Rattanasiri, Mark McEvoy, Colin A Graham, Yuwares Sittichanbuncha, Ammarin Thakkinstian

**Affiliations:** Section for Clinical Epidemiology and Biostatistics, Faculty of Medicine, Ramathibodi Hospital, Mahidol University, 270 Rama VI Road, Toong Phaya Thai, Ratchathewi, Bangkok 10400 Thailand; Department of Emergency Medicine, Faculty of Medicine, Ramathibodi Hospital, Mahidol University, Bangkok, Thailand; Centre for Clinical Epidemiology and Biostatistics, The University of Newcastle, Newcastle, NSW Australia; Accident and Emergency Medicine Academic Unit, Prince of Wales Hospital, The Chinese University of Hong Kong, Sha Tin, New Territories Hong Kong

**Keywords:** ᅟ

## Abstract

**Introduction:**

The aim of this study was to conduct a systematic review and meta-analysis for determining the effects of prehospital adrenaline administration on return of spontaneous circulation, hospital admission, survival to discharge and discharge with cerebral performance category 1 or 2 in out-of-hospital cardiac arrest patients.

**Methods:**

MEDLINE and Scopus databases were searched to identify studies reported to March 2014. Study selection and data extraction were independently completed by two reviewers (PA and SR). The baseline characteristics of each study and number of events were extracted. Risk ratios (RR) and 95% confidence interval (CI) were estimated. Heterogeneity and publication bias were also explored.

**Results:**

In total 15 studies were eligible and included in the study. Of 13 adult observational studies, four to eight studies were pooled for each outcome. These yielded a total sample size that ranged from 2,381 to 421,459. A random effects model suggested that patients receiving prehospital adrenaline were 2.89 times (95% CI: 2.36, 3.54) more likely to achieve prehospital return of spontaneous circulation than those not administered adrenaline. However, there were no significant effects on overall return of spontaneous circulation (RR = 0.93, 95% CI: 0.5, 1.74), admission (RR = 1.05, 95% CI: 0.80, 1.38) and survival to discharge (RR = 0.69, 95% CI: 0.48, 1.00).

**Conclusions:**

Prehospital adrenaline administration may increase prehospital return of spontaneous circulation, but it does not improve overall rates of return of spontaneous circulation, hospital admission and survival to discharge.

**Electronic supplementary material:**

The online version of this article (doi:10.1186/s13054-014-0463-7) contains supplementary material, which is available to authorized users.

## Introduction

Out of hospital cardiac arrest is one of the most urgent Emergency Medical Service (EMS) priorities, in which only 23% of patients survive to hospital admission [1,2], and only 7.6% survive to hospital discharge [1,2]. Factors identified as predictors of survival among out of hospital cardiac arrest patients include the cardiac arrest being witnessed by a bystander, arrest witnessed by EMS personnel, initially shockable cardiac rhythms and bystander cardiopulmonary resuscitation (CPR) [2]. Administration of adrenaline has been a part of advanced life support guidelines for decades [3], for both shockable and non-shockable cardiac rhythms [4-7], to increase cardiac output and preferentially divert blood circulation to the heart and brain during CPR [3]. Animal models indicate that adrenaline administration significantly increases the probability of return of spontaneous circulation (ROSC) [8-11]. However, evidence in humans is limited, with most studies being observational studies with inconsistent results on short term outcomes including ROSC [5,12-18] and hospital admission [5,13,14,16,18-21]. In addition, inconsistent results were also found in long-term outcomes such as one-month survival with good cerebral performance [14,18] and survival to hospital discharge [5,14,16,18-20].

Although three systematic reviews [22-24] have been conducted, a more complete review was still required for the following reasons. One previous review studied a mix of in- and out-of hospital cardiac arrest patients without providing any quantitative conclusion [22], and another systematic review pooled adrenaline effect on survival to hospital discharge but not for prehospital ROSC [23]. Finally, Lin *et al*. conducted a systematic review by including only randomized controlled trials (RCTs), which primarily aimed to compare the standard dosages of adrenaline administration with placebo, vasopressin and high dosage of adrenaline in out of hospital cardiac arrest patients [24]. Although those authors found benefit of adrenaline administration over placebo on ROSC and survival to admission, the results were based on only one RCT. There have been more studies published since these reviews were reported. We, therefore, conducted a systematic review and meta-analysis focused only on out of hospital cardiac arrest patients, which aimed to determine the effects of prehospital adrenaline administration on both short (that is, ROSC, hospital admission) and long term outcomes (that is, survival to discharge and discharged with cerebral performance category (CPC) 1 or 2).

## Methods

### Search strategy

This study followed Meta-Analysis Of Observational Studies in Epidemiology (MOOSE) guidelines [25]. Because we worked on public data, neither approval nor patient consent was required by the Ethics Committee of the Faculty of Medicine, Ramathibodi Hospital, Mahidol University. Two reviewers (PA and SR) identified studies from MEDLINE and Scopus databases since conception to March 2014 using PubMed and Scopus search engines. The following search terms were used: (heart arrest or out of hospital cardiac arrest or ventricular fibrillation or pulseless electrical activity or PEA or asystole or cardiac arrest) and (epinephrine or adrenaline) and (return of spontaneous circulation or ROSC or admission or discharge or death or survival). Studies were firstly selected based on title and abstracts, and full papers were reviewed to make a final decision on selection.

Any type of study (that is, RCT, quasi-experimental study, cohort, or cross sectional study) published in English was selected if it met the following eligibility criteria: studied in patients with out of hospital cardiac arrest, compared clinical outcomes between prehospital adrenaline and no administration, and had at least one of these clinical outcomes of interest: ROSC, hospital admission, survival to hospital discharge, or discharged with CPC 1 or 2. Studies were excluded if there were insufficient data for pooling and if authors did not provide additional data after being contacted twice.

The short-term outcomes of interest included ROSC and hospital admission and the long-term outcomes of interest were survival to discharge and discharged with CPC 1 or 2. The ROSC was defined according to individual original studies as any sign of spontaneous circulation including palpable pulse or measurable blood pressure following CPR [13,16,26,27] occurring during prehospital, at emergency department (ED) arrival, or in-hospital. Survival to discharge referred to patients who were still alive at discharge from hospital. Neurological outcome at discharge was assessed using the Glasgow-Pittsburgh cerebral performance category scale, which categorizes patients as CPC 1 to 5 as follows: good performance, moderate, severe, coma/vegetative, and death, respectively [14,18,26-28].

### Data extraction

Data were extracted by two independent reviewers (PA and SR). Study characteristics (that is, settings, study designs, types of subjects, mean age and percentage of males) were extracted. In addition, clinical data including initial cardiac rhythms, dose and routes of adrenaline administration, presumed cardiac etiologies, witnessed cardiac arrest and bystander CPR status were also extracted. The numbers of subjects who did and did not experience outcomes of interest among adrenaline administration groups were also extracted. Any disagreement was discussed with the senior author (AT).

### Risk of bias assessment

The Newcastle-Ottawa Scale for assessing the quality of a cohort study [29] was modified and used to assess risk of bias for included cohort studies (see Additional file 1: Table S2). This tool consists of five items, which are representative of out of hospital cardiac arrest cohorts, ascertainment of exposure and outcomes, adjusting for confounders and missing data. Each item was graded as low or high risk of bias, and unclear if insufficient information.

### Statistical analysis

The effect of adrenaline administration was estimated for each study using risk ratio (RR) along with its 95% confidence interval (CI). The RR was pooled across studies using the random-effect model if heterogeneity was present [30], otherwise a fixed-effects model was applied. The criteria for declaring heterogeneity was a *P*-value of the Cochrane’s Q statistics <0.1 or I^2^ 25% or higher [31]. Sources of heterogeneity were explored using Galbraith’s plot and meta-regression analysis. Each potential variable (that is, types of subjects, initial cardiac rhythms and proportion of witnessed cardiac arrest) was fitted in a meta-regression model. A sensitivity analysis was applied by excluding candidate studies suspected to be a source of heterogeneity.

Publication bias was assessed using a funnel plot and Harbord’s test [32]. A contour-enhanced funnel plot was applied to explore whether there was any cause of asymmetry due to publication bias or heterogeneity [33]. All analyses were performed using STATA version 12.0. A *P*-value of less than 0.05 was considered as statistically significant, except for the heterogeneity test in which 0.10 was used.

## Results

**Figure 1:**
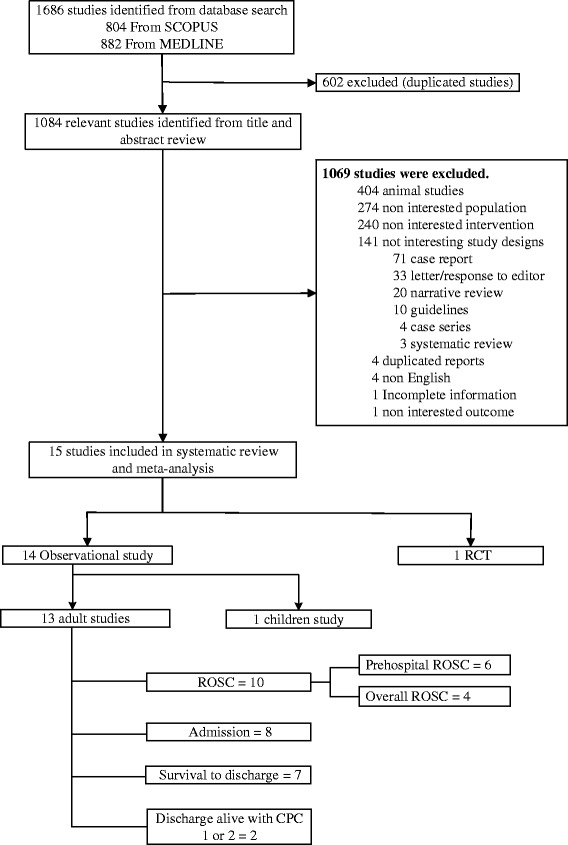
**Flow of selection of studies.**

Eight-hundred and eighty-two studies and 804 studies were identified, respectively, from PubMed and Scopus, see Figure 1. Of these, 602 duplicated studies were excluded, leaving 1,084 studies for screening titles and abstracts. Among them, 1,069 studies did not meet our eligibility criteria and the reasons have been clearly described in Figure 1, leaving 54 studies for review of full papers. Of these, 39 studies were later excluded, which finally resulted in 15 studies for inclusion in pooling data [5,12-21,27,34-36].

The characteristics of the included studies are described in Table 1. Of the 15 eligible studies, 14 were observational studies and only 1 was a RCT [27]. All studies were in adult patients except one which included children [34]. Among 14 adult cohorts, 10 studies [12-18,35-37] reported ROSC, 8 studies [5,13,14,16,18-21] reported admission, 7 studies [5,14,16,18-20,35] reported survival to discharge, and only 2 studies reported discharged with CPC 1 or 2 [14,18]. All studies were EMS based designs, except one study which was hospital-based using a hospital cardiac arrest data registry [18]. Only five studies exclusively included patients with non-trauma [13,14,16,17,35] whereas the others included cohorts in which trauma patients were combined with non-trauma cases [5,12,15,18-21,36]. Patients with specific initial cardiac rhythms were isolated in five studies, which included three pulseless electrical activity (PEA) [14,19,21], one asystole [20] and one ventricular fibrillation [5]. All studies compared administration of a standard dose of adrenaline to no administration. There were four studies [16,17,21,27] which reported the amount of adrenaline administration which ranged from 1 to 5 mg. Since there was only one RCT and one study in children, pooling of the treatment effect versus no administration focused on only thirteen adult cohorts.

**Table 1:** **Description of study and subject characteristics of included studies**

**Author, year [reference]**	**Country**	**Setting**	**Sample size**	**Study design**	**Type of subject**	**Age group**	**Cardiac rhythm**	**Mean age, year**	**Male (%)**	**Cardiac cause (%)**	**Witnessed by bystander (%)**	**Bystander CPR (%)**	**AD (mg)**
Herlitz , 1994 [20]	Sweden	EMS	1,222	Cohort	M	Adult	Asystole	66^a^	69.56	73	50	8.59	-
Dieckmann, 1995 [34]	USA	EMS	65	Cohort	NT	Children	Mixed	1.51	64.62	-	-	-	-
Herlitz, 1995 [5]	Sweden	EMS	1,203	Cohort	M	Adult	VF	70^a^	79.7	-	85	19.7	-
Herlitz, 1995 [19]	Sweden	EMS	748	Cohort	M	Adult	PEA	71^a^	67.11	76	75	8	-
Guyette, 2004 [17]	USA	EMS	298	Cohort	NT	Adult	Mixed	63.5	58.05	-	43.96	28.19	3.8
Ong, 2007 [16]	Singapore	EMS	681	Cohort	NT	Adult	Mixed	63.3	68.75	-	57.1	19.36	1
Vayrynen, 2008 [21]	Findland	EMS	789	Cohort	M	Adult	PEA	66.7	61.85	45.75	56.15	23.07	3.8
Yanagawa, 2010 [15]	Japan	EMS	713	Cohort	M	Adult	Mixed	68	59.89	85	42.5	33.52	-
Jacobs, 2011 [27]	Australia	EMS	534	RCT	M	Adult	Mixed	63.5	72.85	91.39	48.31	51.12	5
Hagihara, 2012 [12]	Japan	EMS	417,188	Cohort	M	Adult	Mixed	70	58.95	55.06	40.33	36.1	-
Hayashi, 2012 [13]	Japan	EMS	3,161	Cohort	NT	Adult	Mixed	73.3	60.2	67.26	100	41.57	-
Machida, 2012 [18]	Japan	Hospital	492	Cohort	M	Adult	Mixed	64	65.85	34.35	33.94	53.05	-
Nordseth, 2012 [14]	Sweden	EMS	174	Cohort	NT	Adult	PEA	75^a^	64.16	56.65	57.23	40.46	-
Neset, 2013 [35]	Sweden	EMS	233	Cohort	NT	Adult	Mixed	74	57.6	56.7	35.7	45.7	-
Goto, 2013 [36]	Japan	EMS	209,577	Cohort	M	Adult	Mixed	68^a^	778.47	86.1	74.89	70.85	-

^a^Median. AD, adrenaline; CPR, cardiopulmonary resuscitation; EMS, emergency medical service; M, mixed trauma and non-trauma; NT, non-trauma; PEA, pulseless electrical activity; RCT, randomized controlled trial; VF, ventricular fibrillation.

*Median.

### Risk of bias assessment

Thirteen adult cohort studies were assessed for risk of bias using the Newcastle-Ottawa scale (see Additional file 1: Table S3). Eleven studies were low risk of bias for representativeness of out of hospital cardiac arrest. There were 11 studies which were graded as low risk of bias for both ascertainment of adrenaline administration and outcomes. Nine studies assessed treatment effects by adjusting confounders, thus they were low risk of confounding bias. Finally, 10 studies were graded as low risk of bias for missing data.

### Return of spontaneous circulation

#### Prehospital ROSC

**Figure 2:**
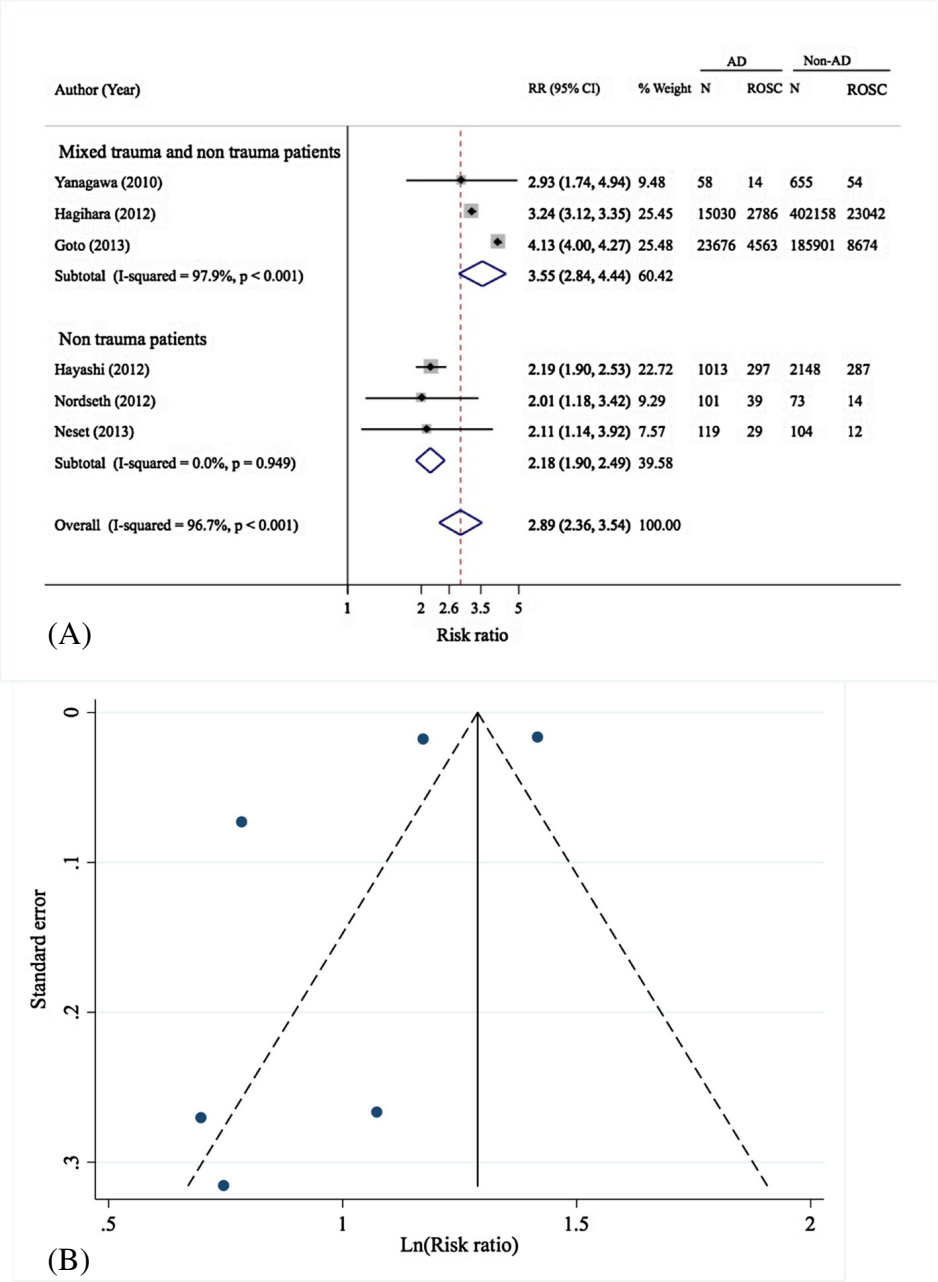
**Forest (A) and funnel plot (B) of pooling of pre-hospital adrenaline effects on prehospital ROSC.** AD, adrenaline; N, number of subjects; Non-AD, non adrenaline; ROSC, return of spontaneous circulation; RR, risk ratio.

Six adult cohorts [12-15,35,36] were included for pooling adrenaline administration effects on prehospital ROSC with sample sizes of 16,321 for adrenaline and 405,138 for non-adrenaline groups, see Table 2. The prehospital adrenaline effect was highly heterogeneous across studies (Q = 153.2, d.f. = 5, *P* <0.001 , I^2^ = 96.7%), see Figure 2A and Table 2. A random-effects model was applied and yielded a pooled RR of 2.89 (95% CI: 2.36, 3.54), indicating that a patient receiving prehospital adrenaline was 2.89 times more likely to experience prehospital ROSC than one not receiving prehospital adrenaline.

**Table 2:** **Pooling effects of prehospital adrenaline on prehospital ROSC**

**Author, year [reference]**	**Sample size**	**Adrenaline group**		**Non-adrenaline group**		**RR (95% CI)**
		**ROSC** ^**a**^	**n**	**ROSC** ^**a**^	**n**	
Yanagawa, 2010 [15]	713	14	58	54	655	2.93 (1.74, 4.94)
Hagihara, 2012 [12]	417,188	2,786	15,030	23.042	402,158	3.24 (3.12, 3.35)
Hayashi, 2012 [13]	3,161	297	1,013	287	2,148	2.19 (1.9, 2.53)
Nordseth, 2012 [14]	174	39	101	14	73	2.01 (1.18, 3.43)
Goto, 2013 [36]	209,577	4,563	23,676	8,674	185,901	4.13 (4.0, 4.27)
Neset, 2013 [35]	223	29	119	12	104	2.11 (1.14, 3.92)
Pooled	421,459	3,165	16,321	23,409	405,138	2.89 (2.36, 3.54)

^a^Prehospital ROSC. CI, confidence interval; n, number; ROSC, return of spontaneous circulation; RR, risk ratio.

Galbraith plot and meta-regression suggested that type of subjects (non-trauma or mixed non-trauma with trauma) and arrest witnessed by bystander might be sources of heterogeneity (data not shown). Thus, a subgroup analysis was performed next by types of subjects (that is, non-trauma only [13,14,35] and mixed trauma and non-trauma cases [12,15,36]). Treatment effect was homogeneous among non-trauma cases (Q = 0, d.f. = 2, *P* <0.95, I^2^ = 0%), but it was still highly varied among mixed cases (Q = 97, d.f. = 2, *P* <0.001, I^2^ = 97.9%). The pooled RRs for these two subgroups were 2.18 (95% CI: 1.9, 2.49) and 3.55, 95% CI: 2.84, 4.44), respectively.

A funnel plot showed a departure from symmetry of the funnel (see Figure 2B), but this was not detected by the Harbord’s test (coefficient = −7.92, *P* = 0.134). However, a contour-enhanced funnel plot was constructed to explore any cause of asymmetry (see Additional file 1: Figure S1). This suggested that all studies fell in the significant area indicating that asymmetry of the funnel was more likely due to missing studies (that is, publication bias) than heterogeneity.

### Overall return of spontaneous circulation

**Figure 3:**
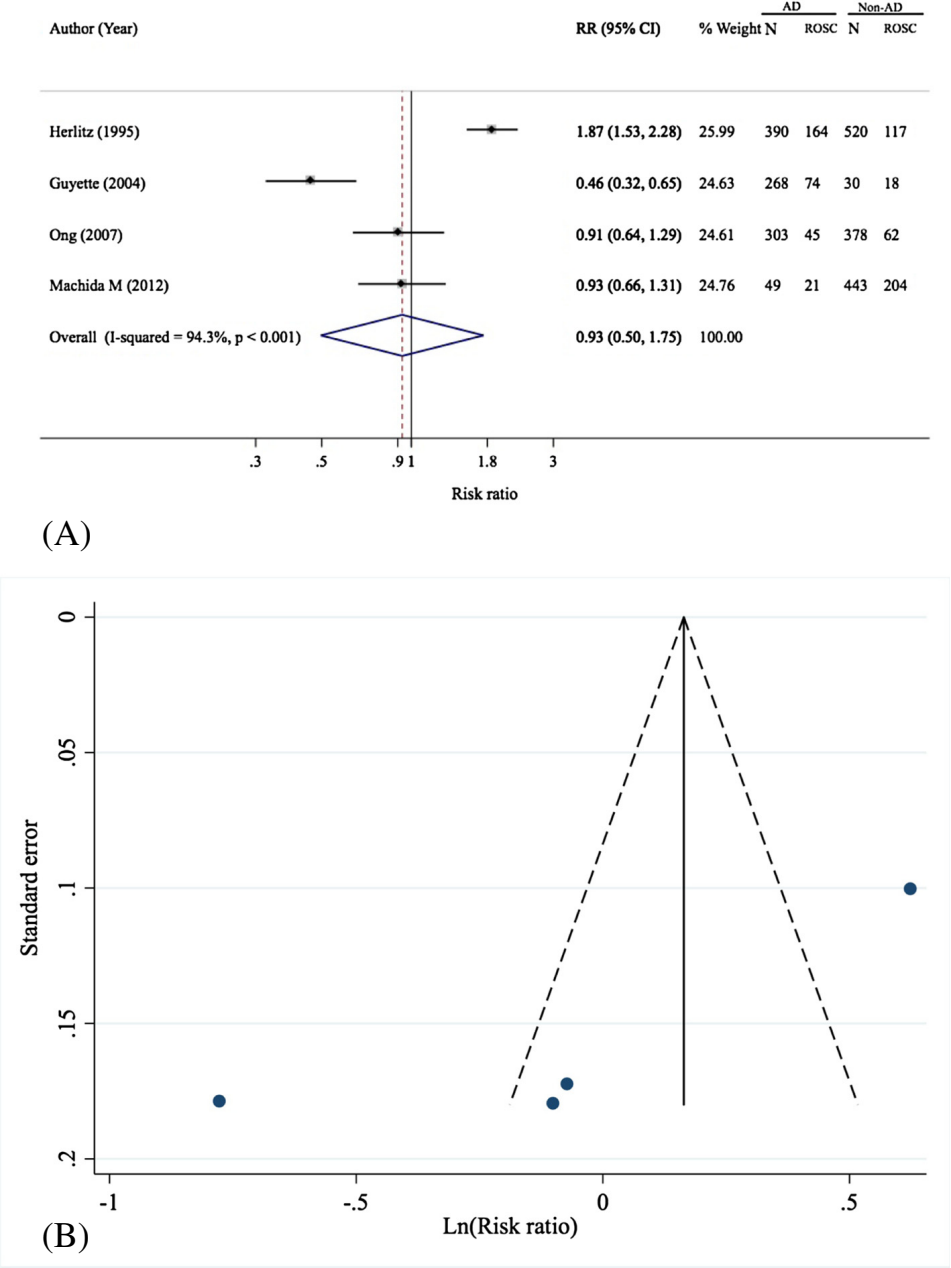
**Forest (A) and funnel plot (B) of pooling of prehospital adrenaline effects on overall ROSC.** AD, adrenaline; N, number of subjects; Non-AD, non adrenaline; ROSC, return of spontaneous circulation; RR, risk ratio.

The effect of prehospital adrenaline on overall ROSC was pooled based on four studies with a total sample size of 2,381 [5,16-18], see Table 3. The adrenaline effect was highly heterogeneous (Q = 52.57, d.f. = 3, *P* <0.001, I^2^ = 94.3%) with a pooled RR of 0.93 (95% CI: 0.5, 1.74), see Table 3 and Figure 3A. This suggested no effect of prehospital adrenaline on achieving overall ROSC. No source of heterogeneity could be identified from Galbraith’s plot or meta-regression analysis.

**Table 3:** **Pooling effects of prehospital adrenaline on overall ROSC**

**Author, year [reference]**	**Sample size**	**Adrenaline group**		**Non-adrenaline group**		**RR (95% CI)**
		**ROSC** ^**a**^	**n**	**ROSC** ^**a**^	**n**	
Herlitz, 1995 [5]	910	164	390	117	520	1.87 (1.53, 2.28)
Guyette, 2004 [17]	298	74	268	18	30	0.46 (0.32, 0.65)
Ong, 2007 [16]	681	45	303	62	378	0.91 (0.64, 1.29)
Machida, 2012 [18]	492	21	49	204	443	0.93 (0.66, 1.31)
Pooled	2,381	304	1,010	401	1,371	0.93 (0.5, 1.75)

^a^Overall ROSC. CI, confidence interval; n, number; ROSC, return of spontaneous circulation; RR, risk ratio.

There was evidence of asymmetry from the funnel plot and the Harbord’s test (coefficient = −9.27, *P* = 0.005), see Figure 3B. The contour enhanced funnel plot showed that 50% of the studies fell in both significant and non-significant areas, indicating heterogeneity was most likely the cause of asymmetry of the funnel (see Additional file 1: Figure S2).

### Hospital admission

**Figure 4:**
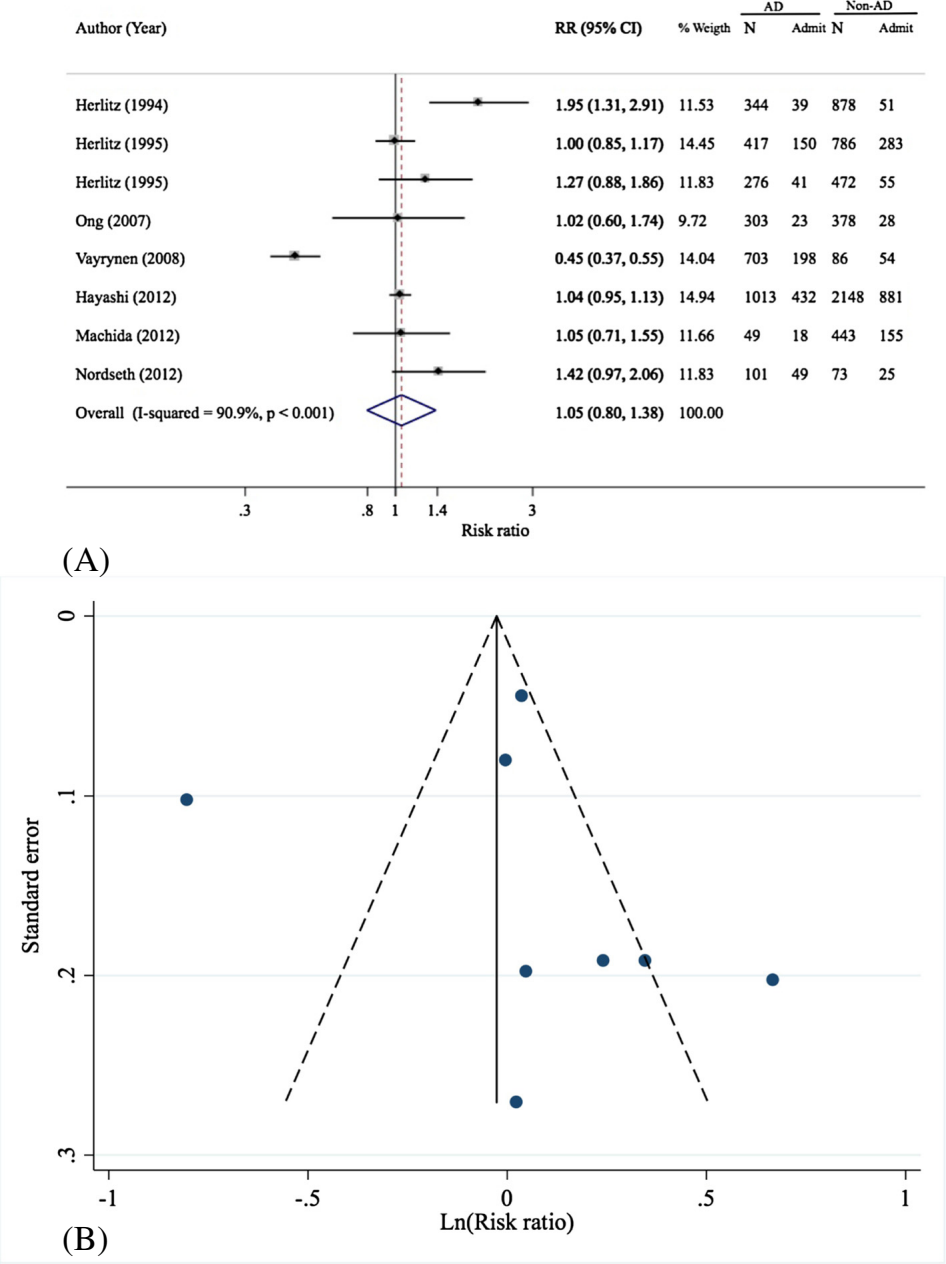
**Forest (A) and funnel plot (B) of pooling of prehospital adrenaline effects on overall hospital admission.** AD, adrenaline; N, number of subjects; Non-AD, non adrenaline; RR, risk ratio.

Eight observational studies assessed the association of prehospital adrenaline administration and hospital admission [5,13,14,16,18-21]. The effect of adrenaline highly varied across studies (Q = 77.08, d.f. = 7, *P* <0.001, I^2^ = 90.9%) with a pooled RR of 1.05 (95% CI:0.80, 1.38), which was not statistically significant, see Table 4 and Figure 4A.

**Table 4:** **Pooling effects of prehospital adrenaline on hospital admission**

**Author, year [reference]**	**Sample size**	**Adrenaline group**		**Non-adrenaline group**		**RR (95% CI)**
		**Admission**	**n**	**Admission**	**n**	
Herlitz, 1994 [20]	1,222	39	344	51	878	1.95 (1.31, 2.91)
Herlitz, 1995 [5]	1,203	150	417	283	786	1.0 (0.85, 1.17)
Herlitz, 1995 [19]	748	41	276	55	472	1.28 (0.88, 1.86)
Ong, 2007 [16]	681	23	303	28	378	1.03 (0.6, 1.74)
Vayrynen, 2008 [21]	789	198	703	54	86	0.45 (0.37, 0.55)
Hayashi, 2012 [13]	3,161	432	1,013	881	2,148	1.04 (0.95, 1.14)
Machida, 2012 [18]	492	18	49	155	443	1.05(0.71, 1.54)
Nordseth, 2012 [14]	174	49	101	25	73	1.42 (0.97, 2.06)
Pooled	8,470	950	3,206	1,532	5,264	1.05 (0.8, 1.38)

A sensitivity analysis was performed by excluding the study [21] which included only non-trauma patients with pulseless electrical activity. The degree of heterogeneity decreased substantially (Q = 12.91, d.f. = 6, *P* = 0.045, I^2^ = 53.5%) and with a pooled RR of 1.15 (95% CI: 1.00, 1.34). Excluding another study [20] which included only patients with asystole did not improve the degree of heterogeneity (Q = 65.01, d.f. =6, *P* <0.001, I^2^ = 90.8%).

The funnel plot showed a little asymmetry of the funnel, which corresponded with the Harbord’s test (coefficient = 0.03, *P* = 0.988), see Figure 4B. A contour-enhanced funnel plot showed that most studies fell in the non-significant area, suggesting that asymmetry was more likely due to heterogeneity (see Additional file 1: Figure S3).

### Survival to discharge

**Figure 5:**
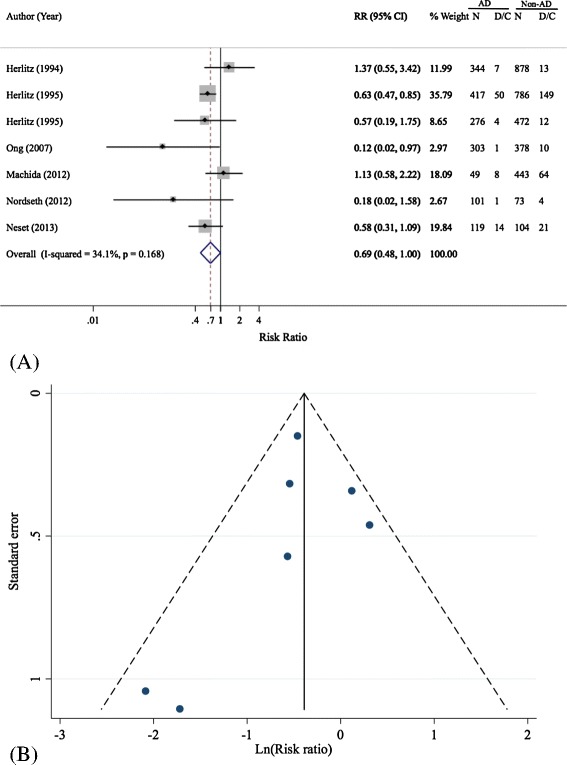
**Forest (A) and funnel plot (B) of pooling of prehospital adrenaline effects on survival to discharge.** AD, adrenaline; D/C, survival to discharge; N, number of subjects; Non-AD, non adrenaline; RR, risk ratio.

Data of seven cohorts [5,14,16,18-20,35] were used for pooling effects of prehospital adrenaline administration on survival to discharge, see Table 5. The pooled RR was 0.69 (95% CI: 0.48, 1.00) with a moderate degree of heterogeneity (Q = 9.1, d.f. = 6, *P* = 0.049, I^2^ = 34.1%), see Table 5 and Figure 5A. This suggested that receiving prehospital adrenaline yielded about a 31% lower chance of discharge alive, but this was of borderline significance. There was no evidence of asymmetry of the funnel as suggested by the Harbord’s test (coefficient = −0.37, *P* = 0.697) and the funnel plot, see Figure 5B.

**Table 5:** **Pooling effects of prehospital adrenaline on discharge alive**

**Author, year [reference]**	**Sample size**	**Adrenaline group**		**Non-adrenaline group**		**RR (95% CI)**
		**Discharge**	**n**	**Discharge**	**n**	
Herlitz, 1994 [20]	1,222	7	344	13	878	1.37 (0.55, 3.42)
Herlitz, 1995 [5]	1,,203	50	417	149	786	0.63 (0.47, 0.85)
Herlitz, 1995 [19]	748	4	276	12	472	0.57 (0.19, 1.75)
Ong, 2007 [16]	681	1	303	10	378	0.13 (0.02, 0.97)
Machida, 2012 [18]	492	8	49	64	443	1.13 (0.58, 2.22)
Nordseth, 2012 [14]	174	1	101	4	73	0.18 (0.02, 1.58)
Neset, 2013 [35]	223	14	119	21	104	0.58 (0.31, 1.09)
Pooled	4,743	85	1,528	74	3,134	0.69 (0.48, 1.00)

## Discussion

We performed a systematic review and meta-analysis to assess the effects of prehospital administration of adrenaline on short-term (that is, ROSC and hospital admission) and long term out of hospital cardiac arrest outcomes (that is, survival to discharge and discharge with CPC 1 or 2) relative to no administration. Pooled treatment effects were performed based on four to eight adult cohorts with total sample sizes of 2,381 to 421,459. Our results indicated that receiving prehospital adrenaline significantly increased prehospital ROSC, but did not increase the overall ROSC, hospital admission, or survival to hospital discharge. However, the treatment effects on prehospital ROSC may be prone to bias from missing studies.

Administration of adrenaline during CPR causes constriction of peripheral vessels, which subsequently increases coronary and cerebral perfusion pressure [3]. Our findings support the effect of adrenaline in increasing prehospital ROSC, which is similar to a previous RCT [27] and systematic reviews by Lin *et al*. [24]. However, our effect size was more precise than the finding by Lin *et al*. because the pooling was based on a larger number of included studies than Lin *et al*., which was based on only one study. In addition, our findings also suggested the adrenaline effect on ROSC in both non-trauma and the mix of trauma and non-trauma cases, although the latter was highly heterogeneous. However, there was no adrenaline effect on overall ROSC, which might be due to variation in the definition and time of ROSC assessment, that is, prehospital, ED, and admission [12,14,18,19,28,36]. Assessing ROSC at the ED or on admission could be very different compared to prehospital ROSC because of the quality of CPR, type of facility, and personnel and equipment used.

No other long term effects (that is, hospital admission and survival to discharge) of prehospital adrenaline were identified. This suggests that adrenaline itself may only have efficacy for inducing prehospital ROSC. Ultimately, survival to hospital discharge is determined more by the underlying clinical condition of the patient; for example, a patient with acute myocardial infarction may be more likely to survive than a patient with end stage respiratory disease, and yet most studies [5,14,18] did not report the underlying diagnoses which leads to difficulties in interpreting the results. In addition, adrenaline is intrinsically a short acting cardiovascular stimulant, which has a limited half-life, and it may be less likely to have a significant effect on long term outcomes for this reason [3]. Assessing treatment effects without adjusting for these factors, as was the case for the included studies, may bias the results.

Our study has a number of strengths. We pooled treatment effects on most relevant clinical short- and long-term outcomes. We attempted to identify the most relevant studies by two independent reviewers using defined search terms and strategies in order to reduce selection bias. Exhaustive searching of the literature resulted in the number of included studies from four to eight cohorts. Although the number of included studies is still small, but, it is larger than a previous systematic review [22], which included five studies [5,16,27,38,39] with different study designs. We explored possible sources of heterogeneity by considering whether a bystander witnessed the arrest, types of subjects and initial cardiac rhythms in a meta-regression. In addition, an attempt was made to distinguish the cause of asymmetry of funnel plots to determine if this was due to heterogeneity or missing studies.

However, our study has some weaknesses. Firstly, our results might be subject to some bias because pooled effects were based on only observational studies, which could not adjust for several known and unknown confounders. The most suitable study design is a RCT with a proper sample size, say approximately 4,500 subjects in total to detect a difference of prehospital ROSC rate of 2.5. Secondly, a subgroup analysis could not be flexibly done because of the small number of included studies. Thirdly, insufficient information on study characteristics resulted in limited exploration of sources of heterogeneity. Finally, we did not pool more relevant clinical outcomes (that is, sustained ROSC and discharge with CPR 1 or 2) because data were not available.

## Conclusions

In summary, prehospital adrenaline administration might increase prehospital ROSC, but not for survival to discharge in out of hospital cardiac arrest patients. However, our findings might be subject to bias from missing studies.

## Key messages

• Currently, there was only one RCT which compared prehospital adrenaline administration versus placebo among out of hospital cardiac arrest patients.

• Receiving prehospital adrenaline significantly increased prehospital ROSC, but not for overall ROSC, hospital admission, and survival to discharge.

## Additional file

Additional file 1
**This file contains Table S1.** Search strategy, **Table S2.** Modified Newcastle-Ottawa quality assessment scale for cohort studies, **Table S3.** Risk of bias assessment for cohort study, **Figure S1.** Contour-enhanced funnel plot of prehospital adrenaline effect on prehospital ROSC, **Figure S2.** Contour-enhanced funnel plot of prehospital adrenaline effect on overall ROSC, **Figure S3.** Contour-enhanced funnel plot of prehospital adrenaline effect on hospital admission, **Figure S4.** Contour-enhanced funnel plot of prehospital adrenaline effect on survival to discharge.

## Abbreviations

AD: adrenaline
95% CI: 95% confidence interval
CPC: cerebral performance category
CPR: cardiopulmonary resuscitation
ED: emergency department
EMS: emergency medical service
M: mixed trauma and non-trauma cases
MOOSE: Meta-analysis Of Observational Studies in Epidemiology
NT: non-trauma cases
OHCA: out of hospital cardiac arrest
PEA: pulseless electrical activity
RCT: randomized controlled trial
ROSC: return of spontaneous circulation
RR: relative risk
VF: ventricular fibrillation
