# Supplementary material for: Effects of prehospital adrenaline administration on out-of-hospital cardiac arrest outcomes: a systematic review and meta-analysis
Source: Crit Care. 2014 Jul 31;18(4):463. doi: 10.1186/s13054-014-0463-7 (PMC4145580; doi:10.1186/s13054-014-0463-7)

**Additional file 1**

**Legend**

**Table S1.** Search strategy

**Table S1.** Search strategy (cont.)

**Table S2.** Modified Newcastle-Ottawa quality assessment scale for cohort stud**ies**

**Table S3.** Risk of bias assessment for cohort study

**Figure S1.** Contour-enhanced funnel plot of pre-hospital adrenaline effect on prehospital ROSC.

**Figure S2.** Contour-enhanced funnel plot of pre-hospital adrenaline effect on overall return of spontaneous circulation

**Figure S3.** Contour-enhanced funnel plot of pre-hospital adrenaline effect on hospital admission

**Figure S4.** Contour-enhanced funnel plot of pre-hospital adrenaline effect on survival to discharge.

**Table S1.** Search strategy

| **Medline (Pubmed) database** | | |
| --- | --- | --- |
| Search | Query | Items |
| #25 | Search ((#11) OR #15) OR #24 | 882 |
| #24 | Search (((((((#16) OR #17) OR #18) OR #19) OR #20) OR #21) OR #23) OR #24 | 1692876 |
| #23 | Search "return of spontaneous circulation" | 1335 |
| #22 | Search ROSC | 1076 |
| #21 | Search "Patient Admission"[Mesh] | 18109 |
| #20 | Search "Patient Discharge"[Mesh] | 18334 |
| #19 | Search Death | 581038 |
| #18 | Search "Death"[Mesh] | 112854 |
| #17 | Search "Survival"[Mesh] | 3857 |
| #16 | Search survival | 1294547 |
| #15 | Search ((#12) OR #13) OR #14 | 130546 |
| #14 | Search "Epinephrine"[Mesh] | 111637 |
| #13 | Search epinephrine | 124562 |
| #12 | Search adrenaline | 130546 |
| #11 | Search (((((((((#1 OR #2) OR #3) OR #4) OR #5 OR #6 OR #7) OR #8) OR #9 OR #10 | 79728 |
| #10 | Search "Heart Arrest"[Mesh] | 33041 |
| #9 | Search "Out-of-Hospital Cardiac Arrest"[Mesh] | 817 |
| #8 | Search "out-of-hospital cardiac arrest" | 2660 |
| #7 | Search "out of hospital cardiac arrest" | 2660 |
| #6 | Search "Ventricular Fibrillation"[Mesh] | 14609 |
| #5 | Search "pulseless electrical activity" | 478 |
| #4 | Search "PEA" | 11010 |
| #3 | Search asystole | 47639 |
| #2 | Search "ventricular fibrillation" | 21832 |
| #1 | Search "cardiac arrest" | 20803 |

**Table S1.** Search strategy (continue)

| **Scopus database** |
| --- |
| ((TITLE-ABS-KEY("out of hospital cardiac arrest")) OR (TITLE-ABS-KEY("cardiac arrest")) OR (TITLE-ABS-KEY("ventricular fibrillation")) OR (TITLE-ABS-KEY("asystole")) OR (TITLE-ABS-KEY("heart arrest")) OR (TITLE-ABS-KEY("pulseless electrical activity")) OR (TITLE-ABS-KEY("PEA"))) AND ((TITLE-ABS-KEY("epinephrine")) OR (TITLE-ABS-KEY("adrenaline"))) AND ((TITLE-ABS-KEY("return of spontaneous circulation")) OR (TITLE-ABS-KEY(rosc)) OR (TITLE-ABS-KEY(death)) OR (TITLE-ABS-KEY(survival)) OR (TITLE-ABS-KEY(discharge)) OR (TITLE-ABS-KEY(admission))) |

**Table S2.** Modified Newcastle-Ottawa quality assessment scale for cohort stud**ies**

| **Items** | | **Risk of bias** |
| --- | --- | --- |
| Representativeness of the OHCA cohort | |  |
|  | True representativeness of OHCA | Low |
|  | Partial Representativeness of OHCA | High |
|  | No description | Unclear |
| Ascertainment of exposure | |  |
|  | Secure record or structured interview | Low |
|  | Written self report | High |
|  | No description | Unclear |
| Assessment of outcome | |  |
|  | Independent blind assessment or record linkage | Low |
|  | Self report | High |
|  | No description | Unclear |
| Adjusted analysis for confounders | |  |
|  | Yes | Low |
|  | No | High |
| Missing data | |  |
|  | Less than 5% | Low |
|  | More than 5% | High |
|  | No statement | Unclear |

OHCA; out of hospital cardiac arrest

**Table S3.** Risk of bias assessment for cohort study

| Author, Year (reference) | Representativeness of OHCA | Ascertainment of AD | Ascertainment of outcomes | Adjusted analysis | Missing data |
| --- | --- | --- | --- | --- | --- |
| Herlitz, 1994 [20] | Low | Unclear | Unclear | High | Low |
| Herlitz, 1995 [5] | High | Unclear | Unclear | High | High |
| Herlitz, 1995 [19] | Low | Unclear | Unclear | Low | Low |
| Guyette, 2004 [17] | Low | Low | Low | Low | Low |
| Ong, 2007 [16] | Low | Low | Low | Low | Low |
| Vayrynen, 2008 [21] | Low | Low | Low | Low | Low |
| Yanagawa, 2010 [15] | Low | Low | Low | Low | Low |
| Hagihara, 2012 [12] | Low | Low | Low | Low | Low |
| Hayashi, 2012 [13] | Low | Low | Low | Low | Low |
| Machida, 2012 [18] | High | Low | Low | High | Unclear |
| Nordseth, 2012 [14] | Low | Low | Low | High | High |
| Neset, 2013 [35] | Low | Low | Low | Low | Low |
| Goto, 2013 [36] | Low | Low | Low | Low | Low |

AD; adrenaline, OHCA; out of hospital cardiac arrest

**Figure S1.** Contour-enhanced funnel plot of pre-hospital adrenaline effect on prehospital ROSC.


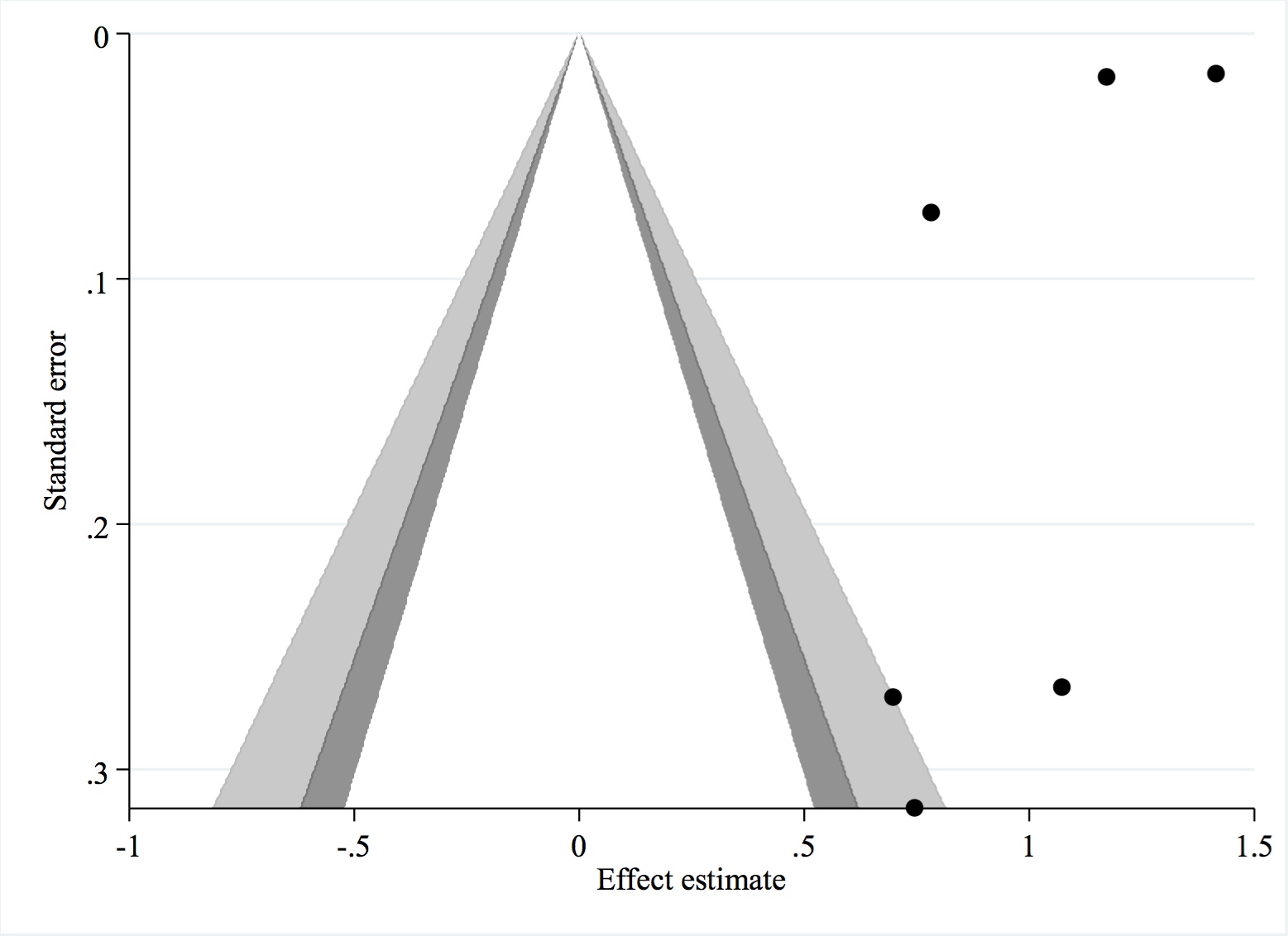


**Figure S2.** Contour-enhanced funnel plot of pre-hospital adrenaline effect on overall return of spontaneous circulation


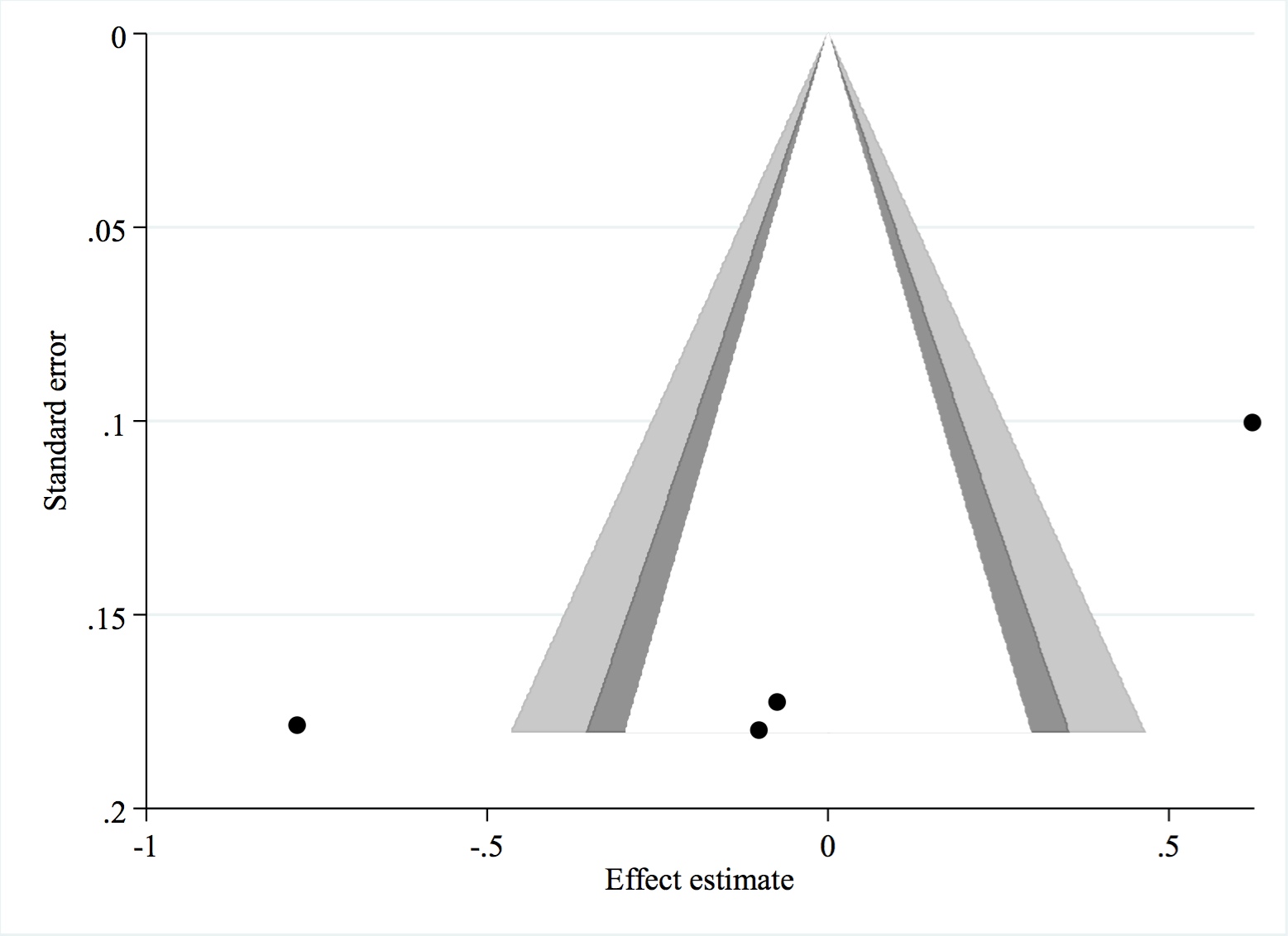


**Figure S3.** Contour-enhanced funnel plot of pre-hospital adrenaline effect on hospital admission


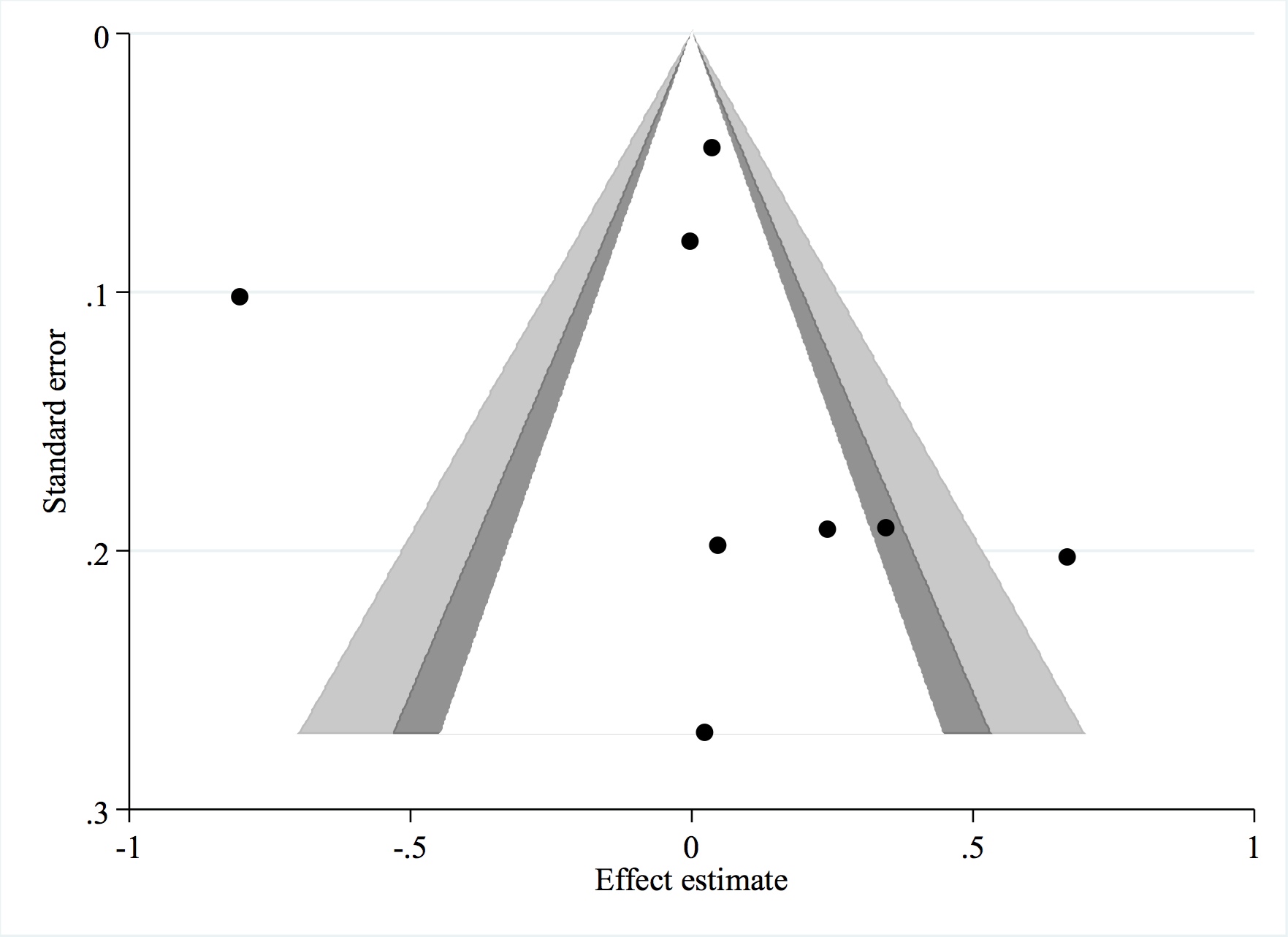


**Figure S4.** Contour-enhanced funnel plot of pre-hospital adrenaline effect on survival to discharge.


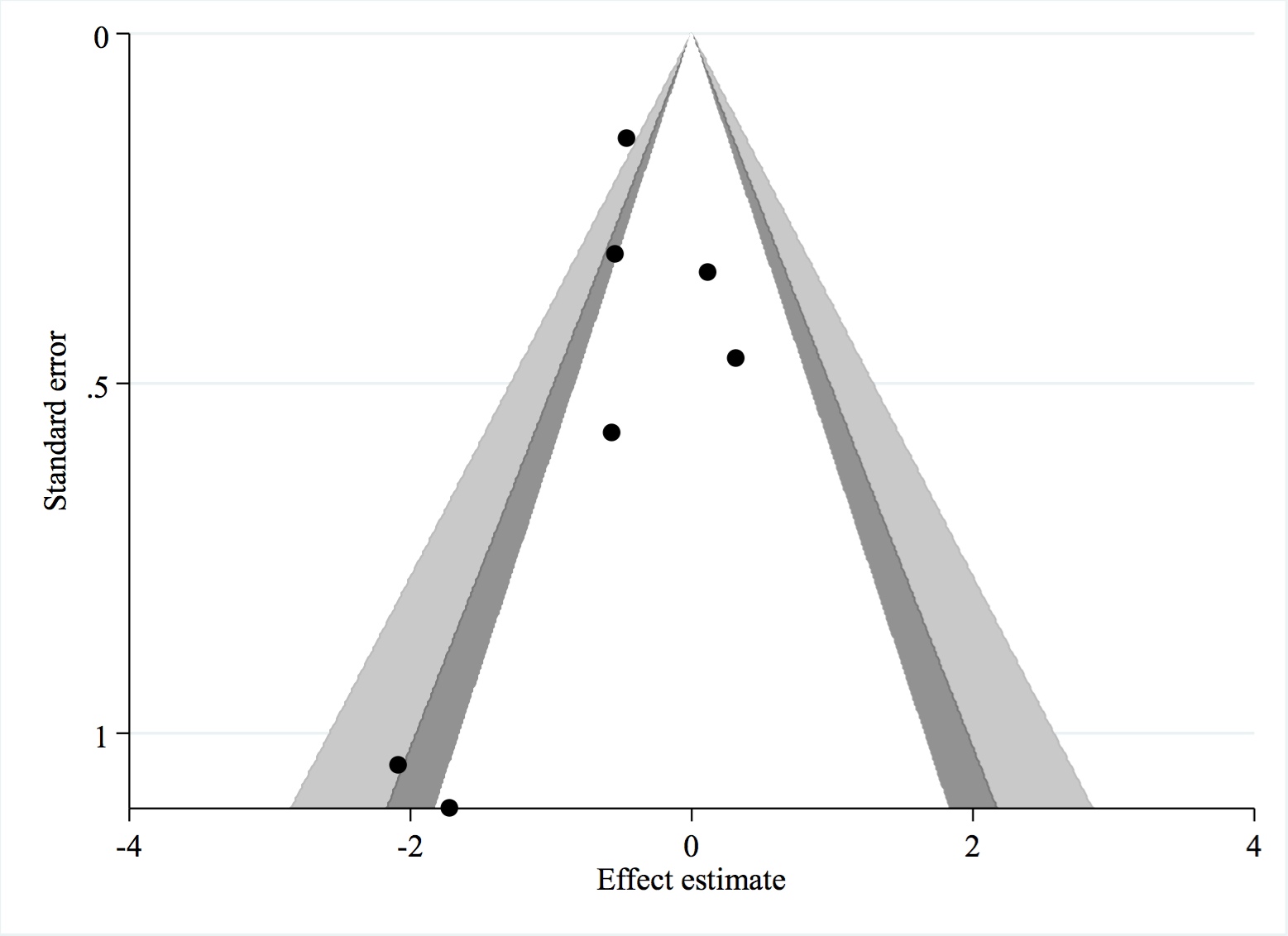

Supplement: Additional file 1 — This file contains Table S1. Search strategy, Table S2. Modified Newcastle-Ottawa quality assessment scale for cohort studies, Table S3. Risk of bias assessment for cohort study, Figure S1. Contour-enhanced funnel plot of prehospital adrenaline effect on prehospital ROSC, Figure S2. Contour-enhanced funnel plot of prehospital adrenaline effect on overall ROSC, Figure S3. Contour-enhanced funnel plot of prehospital adrenaline effect on hospital admission, Figure S4. Contour-enhanced funnel plot of prehospital adrenaline effect on survival to discharge. [file 13054_2014_463_MOESM1_ESM.docx]
